# Safety first! An audit to improve airway protection during student placement of preformed metal crowns

**DOI:** 10.1038/s41415-025-8493-x

**Published:** 2025-08-08

**Authors:** Danielle Stevenson, Paula Waterhouse, Helen J. Rogers

**Affiliations:** 41415402196001 Newcastle Dental Hospital, Newcastle-upon-Tyne Hospitals NHS Foundation Trust, UK; School of Dental Sciences, Faculty of Medical Sciences, Newcastle University, UK; 41415402196002 School of Dental Sciences, Faculty of Medical Sciences, Newcastle University, UK

## Abstract

**Introduction** The Hall technique is an evidenced-based approach to managing primary caries. Airway protection (AP) is essential when placing preformed metal crowns (PMCs), preventing aspiration/ingestion.

**Aim** Assess undergraduates' compliance in AP when placing PMCs.

**Standard** Conforming with guidance from the University of Dundee, 100% of undergraduates will use the following AP during PMC placement: 1) child seated upright (not reclined greater than 45^o^); 2) use of a gauze swab square or Micro-Stix applicator for the entire procedure.

**Methods** Data were obtained prospectively via a standardised *pro forma* completed by calibrated clinical teachers observing student placement of PMCs. Clinical teachers were advised to intervene if they had safety concerns. Data were collected via convenience sample of 50 PMCs per cycle.

**Results** In Cycle 1, 15 PMC placements met the standard, with nine episodes of intervention. A new AP protocol was introduced and embedded within the curriculum. In Cycle 2, 46 PMCs met the standard, with three episodes of intervention.

**Discussion** Despite an improvement in AP of 62%, further changes are recommended to reach the 100% standard set.

**Conclusion** A comprehensive action plan including an AP protocol and teaching can improve compliance with guidance on PMC placement. Further training is proposed to meet the standard.

**Supplementary Information:**

Zusatzmaterial online: Zu diesem Beitrag sind unter 10.1038/s41415-025-8493-x für autorisierte Leser zusätzliche Dateien abrufbar.

## Key points

- Highlights the importance of airway protection during placement of preformed metal crowns.
- Demonstrates how an airway protection protocol can improve student compliance with safety precautions during placement of preformed metal crowns.
- Outlines a whole team approach to addressing a clinical challenge.

**Supplementary Information:**

Zusatzmaterial online: Zu diesem Beitrag sind unter 10.1038/s41415-025-8493-x für autorisierte Leser zusätzliche Dateien abrufbar.

## Introduction

The Hall technique offers a biological approach to managing carious primary teeth, whereby a preformed metal crown (PMC) is cemented over the tooth without prior removal of the caries or administration of local anaesthetic.^1^ While careful case selection is warranted, there is a wealth of evidence to demonstrate the effectiveness and efficacy of this approach, with success rates of 97% at five years and high levels of acceptance from children.^2^^,^^3^^,^^4^^,^^5^^,^^6^ This evidence-based approach has been taught to undergraduate dentistry and dental therapy students at Newcastle University for over ten years, and has now been adopted by all dental schools in the United Kingdom.

The Hall technique is considered a safe procedure, though the placement of a PMC poses a risk of accidental ingestion or aspiration. Foreign body aspiration or ingestion is rare, accounting for 1-12% of all patient safety incidents in dentistry, with ingestion occurring more frequently than aspiration.^7^^,^^8^ Nonetheless, when these incidents do occur, they may be associated with high levels of morbidity and can be life-threatening.^8^^,^^9^ Many ingested dental foreign bodies can pass through the gastrointestinal tract without the need for intervention; although, endoscopic removal has been reported to be required in up to 43% of cases.^10^ Inhaled foreign bodies necessitate removal; smaller objects left *in situ* may remain asymptomatic for many months before causing complications, while larger objects can cause obstruction, requiring management as a medical emergency.^10^ Further to patient-related morbidity, these incidents can be stressful for dental teams to manage and may incur legislative consequences.^8^ There is only one published case report of an aspirated PMC in a healthy young child who was receiving treatment under sedation,^11^ though the true incidence of aspiration events in children is likely to be under-reported.

Prevention of foreign body ingestion or aspiration must be prioritised when placing PMCs in children, hence precautions are advised in the Hall technique user guide.^1^ These include ensuring the child is not reclined more than 45 degrees in the dental chair and using physical airway protection (AP), such as a gauze swab, to catch any free objects, or using adhesive applicators to hold onto the PMC.^1^ These precautions should be maintained throughout the procedure, which often involves ‘trying in' a PMC to check the size before cementation and can sometimes require speedy removal of the PMC if seated incorrectly. Furthermore, unexpected patient movements, which commonly occur in young children, can increase the risk of accidental ingestion or aspiration,^10^ so the clinician must assess whether the child is considered a suitable candidate for PMCs.

Several undergraduate dentistry and dental therapy students at Newcastle University were observed to have not taken sufficient precaution to protect the airway. The evidence suggests that foreign body inhalation and aspiration incidents occur more commonly with inexperienced practitioners, which highlights the need for students to be particularly vigilant.^10^ This which prompted the present audit to be undertaken.

This audit aimed to prospectively assess undergraduate students' measures for AP while placing PMCs. The gold standard for this audit was derived from the recommendations within the Hall technique user guide,^1^ requiring that 100% of students:

- Sit the child upright (not reclined by more than 45 degrees in the dental chair) and
- Use either a gauze swab square or a Micro-Stix applicator to protect the airway for the entire procedure (from ‘try-in' to satisfactory cementation, where removal with a bur is not required).

These materials are available on the paediatric dental clinic for students to use during PMC placement.

Further to assessing current compliance with this standard, this audit aimed to determine if improvements to care were required to optimise paediatric patient safety during this procedure and to implement and evaluate any necessary changes.

## Methods

Before starting the audit, the project was registered on the Newcastle upon Tyne Hospitals NHS Foundation Trust website on 1/4/21 (project number 17134). This manuscript has been written in line with the Standards for Quality Improvement Reporting Excellence in Education **(**SQUIRE-EDU) guidelines.^12^

### Questionnaire development

An AP questionnaire (see online Supplementary Information) was designed for data collection using the defined set standards, after a pilot questionnaire was trialled. The pilot questionnaire was trialled by five clinical teachers and feedback was sought from each. Modifications were made to the questionnaire before data collection.

### Training and calibration

Training was provided for the clinical teachers who would oversee students placing PMCs. This involved showing each clinical teacher a picture of what was meant by sitting a child upright, where the child is not reclined by more than 45 degrees in the dental chair. Methods of AP were discussed with the clinical teaching team during the training, including how to place a gauze swab square in a patient's mouth and how to use a Micro-Stix applicator correctly. Clinical teachers were advised to intervene when supervising the procedure if they had any safety concerns. Various clinical scenarios were used to calibrate clinical teachers in completing the questionnaire appropriately.

### Data collection

A total of 50 AP questionnaires were completed prospectively by clinical teachers overseeing the placement of PMCs using the Hall technique on a child patient by undergraduate students in the paediatric department at Newcastle Dental Hospital. It was concluded that a sample size of 50 questionnaires would be large enough to be representative of the data to be studied. No patient or student details were taken during this audit to ensure anonymity was maintained. Completion of the questionnaire implied consent.

The first cycle of the audit took place between 17 May 2021 and 2 July 2021. Following the first cycle, the results were discussed with the department and an action plan was developed. Changes were implemented before a second cycle, which took place between 24 October 2022 and 6 January 2023. Further discussion of Cycle 2 results was undertaken within the department and further actions were considered.

### Data analysis

Data from the questionnaires were inputted and collated using a Microsoft Excel (Microsoft Corporation, Washington, USA) spreadsheet and simple descriptive statistics were used to summarise the findings.

## Results

A total of 50 AP questionnaires were completed for each cycle, respectively.

### Cycle 1 

**Fig. 1:**
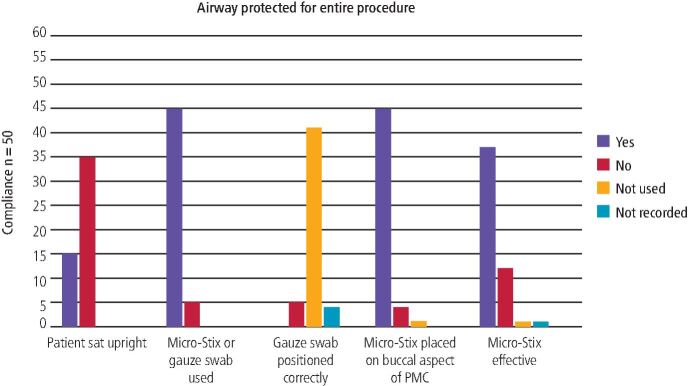
Compliance with the set standard relating to question three, four and five of the AP questionnaire in Cycle 1

In Cycle 1, notable deficiencies were identified regarding undergraduate students' AP during PMC placement (Fig. 1).

Only 15 PMCs (30%) were placed with the child sat upright, with nine episodes where clinical teachers were required to intervene. Of the 35 PMCs where the standard was not met (70%), one clinical teacher commented that they could not sit the child upright to place the PMC, as they could not see what they were doing. Another clinical teacher mentioned that they could not sit the child upright as they were providing treatment for a very tiny child.

On five occasions (10%), a gauze swab square was used to protect the airway (Table 1). In all of these, the gauze was positioned incorrectly, and one clinician intervened for patient safety. On one occasion (*n* = 50) (2%) micropore tape was used on the buccal aspect of the PMC to protect the airway instead of a Micro-Stix applicator or a gauze swab square because no Micro-Stix applicators were available.

**Table 1:** Compliance with question three, four and five of the AP questionnaire during Cycle 1, including whether the clinician intervened for patient safety

**Standards**	**Compliance n/50 (%)**						
	**Yes (%)**	**No (%)**	**Not used (%)**	**Not recorded (%)**	**Clinician intervention for patient safety**		
					**Yes (%)**	**No (%)**	**Not recorded (%)**
Gauze swab positioned correctly for the entire procedure	0 (0)	5 (10)	41 (82)	4 (8)	1 (2)	11 (22)	38 (76)
Micro-Stix placed on buccal aspect of PMC	45 (90)	4 (8)	1 (2)	0 (0)	5 (10)	28 (56)	17 (34)
Micro-Stix effective for entire procedure	36 (72)	12 (24)	1 (2)	1 (2)	9 (18)	28 (56)	13 (26)

On 49 occasions (98%), a Micro-Stix applicator was used to protect the airway during the placement of a PMC and in 45 (92%) of these, the Micro-Stix applicator was used for the entire procedure. Of the four instances (8%) where a Micro-Stix applicator was not used for the entire procedure, two Micro-Stix applicators had been removed before seating the PMC, and on two occasions, the PMCs had to be removed and re-seated, due to being positioned incorrectly. A comment was left by the clinical teacher explaining that there was no form of AP used while removing and re-seating the crown, on those two occasions. On all four occasions, the clinical teachers intervened for patient safety.

The Micro-Stix applicator was placed on the occlusal aspect of the PMC in four instances (8%). The students were asked to reposition the Micro-Stix applicator on the buccal aspect of the PMC.

Micro-Stix applicators were effective for the entire procedure during placement of 36 PMCs (73%). Of the 12 occasions where the Micro-Stix applicators were not effective for the entire procedure, the most common reported reason for failure was loss of adhesion between PMC try-in and placement.

The first cycle of this audit successfully highlighted that the gold standard for AP when placing PMCs at an undergraduate clinic was not met.

### Action plan 1

Cycle 1 was presented and discussed with all members of the paediatric dental team at the departmental clinical governance afternoon in February 2022 and an action plan and AP protocol was developed.

The AP protocol (see online Supplementary Information) was embedded into all teaching on PMCs for undergraduate programmes across all stages. Updated AP teaching covered how to use micropore tape correctly to protect the airway and advised students to use one Micro-Stix applicator on each PMC rather than multiple PMCs to prevent Micro-Stix applicators losing their adhesion. Students were reminded that they could only remove the AP once the clinical teacher had confirmed that the PMC was seated correctly.

A PMC AP sticker (see online Supplementary Information) was introduced to improve documentation for this procedure to use in the paper-based dental records and to serve as a reminder to students. A presentation of the audit findings, action plan and AP protocol was emailed to every clinical teacher before the start of the academic year in September 2022.

### Cycle 2

**Fig. 2:**
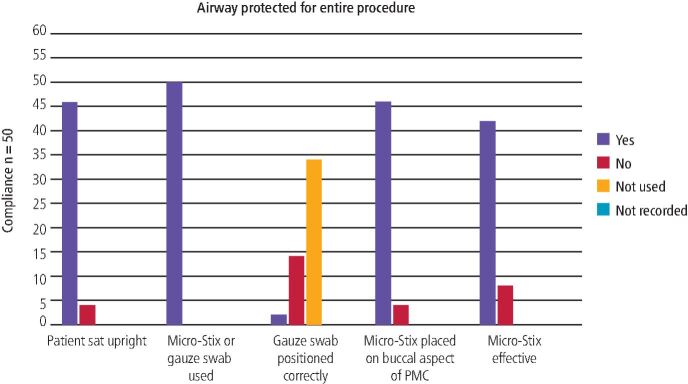
Compliance with the set standard for question three, four and five of the AP questionnaire in Cycle 2

**Fig. 3:**
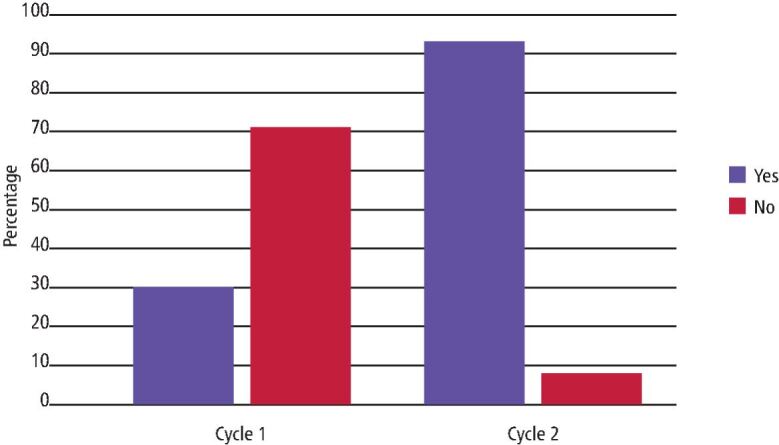
A comparison of compliance with AP standard between Cycle 1 and Cycle 2

Cycle 2 found 46 PMCs (92%) met the standard, which was a substantial increase of 62% in compliance from the first cycle (Table 2, Fig. 2, Fig. 3). Clinical teachers intervened on three out of the four occasions where the standard was not met. On the occasion when the clinical teacher did not intervene, a comment was recorded explaining that it was not possible to sit the patient upright as their behaviour was challenging.

**Table 2:** Compliance with AP standard in Cycle 1 and Cycle 2, including whether the clinical teacher intervened for patient safety

**Standards**	**Compliance n/50 (%)**									
	**Cycle 1**					**Cycle 2**				
	**Yes (%)**	**No (%)**	**Clinician intervention for patient safety**			**Yes (%)**	**No (%)**	**Clinician intervention for patient safety**		
			**Yes (%)**	**No (%)**	**Not recorded (%)**			**Yes (%)**	**No (%)**	**Not recorded (%)**
Patient sat upright for the entire procedure	15 (30)	35 (70)	9 (18)	35 (70)	6 (12)	46 (92)	4 (8)	3 (6)	31 (62)	16 (32)
Micro-Stix or gauze swab used for the entire procedure	45 (90)	5 (10)	4 (8)	29 (58)	17 (34)	50 (100)	0 (0)	0 (0)	30 (60)	20 (40)

Appropriate AP (Micro-Stix applicator or gauze swab square) was used in 100% of PMC placements; an increase in 8% from Cycle 1. The gauze swab square was selected for AP more frequently in Cycle 2 compared to Cycle 1. The gauze swab square was positioned correctly in only two of the 16 occasions when it was used (Table 3). For the 14 occasions (88%) where the gauze swab square was positioned incorrectly, eight episodes reported clinical teacher intervention.

**Table 3:** Compliance with question three, four and five of the AP questionnaire during Cycle 2, including whether the clinician intervened for patient safety

**Standards**	**Compliance n/50 (%)**						
	**Yes (%)**	**No (%)**	**Not used (%)**	**Not recorded (%)**	**Clinician intervention for patient safety**		
					**Yes (%)**	**No (%)**	**Not recorded (%)**
Gauze swab positioned correctly for the entire procedure	2 (4)	14 (28)	34 (68)	0 (0)	0 (0)	11 (22)	39 (78)
Micro-Stix placed on buccal aspect of PMC	46 (92)	4 (8)	0 (0)	0 (0)	3 (6)	29 (58)	18 (36)
Micro-Stix effective for entire procedure	42 (84)	8 (16)	0 (0)	0 (0)	7 (14)	25 (50)	18 (36)

Similarly to Cycle 1, of the 50 episodes of PMC placement in Cycle 2, there were four instances (8%) where students placed the Micro-Stix applicator on the occlusal aspect of the PMC. Of these four instances, intervention by a clinical teacher was reported three times. Most (*n* = 42; 84%) of the Micro-Stix applicators placed in Cycle 2 were effective for the entire procedure, which was an increase from Cycle 1 (*n* = 36; 73%). Three comments highlighted that the Micro-Stix applicator was replaced during the procedure due to adhesion failure. Intervention for patient safety was required on seven occasions when the Micro-Stix applicator proved ineffective for use during the entire procedure.

### Action plan 2

The findings from Cycle 2 were discussed and feedback was sought from the paediatric department, and it was agreed that the following actions would be undertaken:

- A recorded demonstration of optimal AP for students and clinical teachers was created to enhance our clinical teaching, for both staff and students
- Hands-on AP teaching was delivered to all clinical teachers at a staff training day to revise and ensure that everybody was on board with the updated AP protocol. Staff were reminded at this training session that they must intervene if they are not confident with a student's AP during PMC placement
- Compare all forms of AP during PMC placement to evaluate reliability of adhesion, cost-effectiveness and sustainability.

A third cycle of the audit is planned after the current academic year to further assess compliance with the revised AP action plan.

## Discussion

This audit demonstrates a substantial improvement in undergraduate student AP during placement of PMCs following implementation of a comprehensive action plan. This addressed concerns raised by staff regarding airway safety of child patients undergoing this common procedure.

A key feature of the action plan was the development and implementation of an AP protocol. The literature suggests that a procedural protocol with which the whole team are familiar (not just the treating clinician) has the potential to reduce patient safety incidents by acknowledging human factors.^13^ The benefits can be two-fold: firstly, by acting as an *aide-memoire* to ensure critical steps are not missed, but also by improving communication and streamlining interaction between members of the clinical team.^13^ It should be acknowledged that children who cannot tolerate treatment as per this protocol should instead be treated by an experienced clinician, outwith the undergraduate training programme.

It is important to recognise that the action plan implemented did not achieve the standard specified in the Hall technique user guide,^1^ hence further improvements are necessary. One potential factor limiting the success of the action plan implemented before Cycle 2 may be that the teaching delivered to clinical teachers was provided via a pre-recorded lecture. There is evidence to suggest that learner engagement fluctuates during a pre-recorded lecture and individuals may find it challenging to retain content.^14^ As such, an in-person tutorial may have provided a more impactful approach to sharing this information.

This audit has a number of strengths. To the authors' knowledge, this is the first audit to address airway safety in children during PMC placement, and takes a novel perspective by focusing on undergraduate students' compliance with the standards outlined in the user guide.^1^ The prospective approach to audit is infrequently used as it is more resource-intensive than retrospective audit, yet provides an opportunity to gather first-hand, detailed information, without reliance on record-keeping.^15^ A further strength relates to the involvement of the whole team in the development and implementation of an action plan following each cycle. This approach ensured a range of perspectives were incorporated, potentially improving ‘buy-in' from colleagues to support implementation of the proposed changes.

The authors acknowledge that other forms of AP are available beyond the approaches discussed in this paper. Given the increased emphasis on sustainability in healthcare, future research is required to explore the environmental impact of these approaches, particularly the Micro-Stix applicator, whereby more than one applicator was required during a single PMC placement due to loss of adhesion. A reduction in single-use plastic would help the NHS achieve its commitment to improve sustainability.^16^

## Conclusion

AP should be prioritised during placement of Hall technique PMCs. This audit demonstrates a comprehensive approach to improve compliance with AP standards when undergraduate dentistry and dental therapy students undertake this procedure for young children. Further action is required to obtain optimal compliance.

## Supplementary Information

Supplementary Information (PDF 248KB)

## Data Availability

Data are available upon reasonable request to the authors.

## References

[1] Innes N, Evans D. *The Hall Technique: a Child Centred Approach to Managing the Carious Primary Molar. A Users Manual*. 4th ed. Dundee: University of Dundee, 2015.

[2] Bell S J, Morgan A G, Marshman Z, Rodd H D. Child and parental acceptance of preformed metal crowns. *Eur Arch Paediatr Dent* 2010; **11:** 218-224. 10.1007/BF03262750 20932394

[3] Page L A, Boyd D H, Davidson S E, McKay S K, Thomson W M, Innes N P. Acceptability of the Hall technique to parents and children. *N Z Dent J* 2014; **110:** 12-17. 24683915

[4] Innes N P T, Evans D J P, Bonifacio C C *et al*. The Hall technique 10 years on: questions and answers. *Br Dent J* 2017; **222:** 478-483. 10.1038/sj.bdj.2017.273 28336976

[5] Innes N P T, Evans D J P, Stirrups D R. Sealing caries in primary molars: randomized control trial, 5-year results. *J Dent Res* 2011; **90:** 1405-1410. 10.1177/0022034511422064 21921249

[6] Tedesco T K, Innes N P, Gallegos C L *et al*. Success rate of Hall technique for restoring carious primary molars: systematic review and meta-analysis. *Evid Based Dent* 2025; **26:** 65-66. 10.1038/s41432-024-01044-0 39152338

[7] Ensaldo-Carrasco E, Suarez-Ortegon M F, Carson-Stevens A, Cresswell K, Bedi R, Sheikh A. Patient safety incidents and adverse events in ambulatory dental care: a systematic scoping review. *J Patient Saf* 2021; **17:** 381-391. 10.1097/PTS.0000000000000316 27611771

[8] Tiwana K K, Morton T, Tiwana P S. Aspiration and ingestion in dental practice: a 10-year institutional review. *J Am Dent Assoc* 2004; **135:** 1287-1291. 10.14219/jada.archive.2004.0404 15493393

[9] Cameron S M, Whitlock W L, Tabor M S. Foreign body aspiration in dentistry: a review. *J Am Dent Assoc* 1996; **127:** 1224-1229. 10.14219/jada.archive.1996.0415 8803399

[10] Huh J, Lee N, Kim K-Y *et al*. Foreign body aspiration and ingestion in dental clinic: a seven-year retrospective study. *J Dent Anesth Pain Med* 2022; **22:** 187-195. 10.17245/jdapm.2022.22.3.187 PMC9171336 35693354

[11] Adewumi A, Kays D W. Stainless steel crown aspiration during sedation in pediatric dentistry. *Pediatr Dent* 2008; **30:** 59-62. 18402101

[12] Ogrinc G, Armstrong G E, Dolansky M A, Singh M K, Davies L. SQUIRE-EDU (Standards for QUality Improvement Reporting Excellence in Education): publication guidelines for educational improvement. *Acad Med* 2019; **94:** 1461-1470. 10.1097/ACM.0000000000002750 PMC6760810 30998575

[13] Calland J F, Guerlain S, Adams R B, Tribble C G, Foley E, Chekan E G. A systems approach to surgical safety. *Surg Endosc* 2002; **16:** 1005-1015. 10.1007/s00464-002-8509-3 12000985

[14] Davidesco I, Glaser N, Stevenson I H, Dagan O. Detecting fluctuations in student engagement and retention during video lectures using electroencephalography. *Br J Educ Technol* 2023; **54:** 1895-1916.

[15] Benjamin A. Audit: how to do it in practice. *BMJ* 2008; **336:** 1241-1245. 10.1136/bmj.39527.628322.AD PMC2405828 18511799

[16] NHS England. Delivering a ‘net zero' National Health Service. 2022. Available at https://www.england.nhs.uk/greenernhs/publication/delivering-a-net-zero-national-health-service/ (accessed 25 May 2024).

