# Supplementary material for: Safety first! An audit to improve airway protection during student placement of preformed metal crowns
Source: Br Dent J. 2025 Aug 8;239(3):204–8. doi: 10.1038/s41415-025-8493-x (PMC12334350; doi:10.1038/s41415-025-8493-x)
Supplement: Supplementary file 1 — Supplementary Information (PDF 248KB) [file 41415_2025_8493_MOESM1_ESM.pdf]

Supplementary material

1. Airway protection questionnaire

- Please complete the following questionnaire for any student providing a preformed metal crown (PMC) using the Hall Technique on the Paediatric clinic.
- Please place a tick in the relevant yes or no boxes for all questions. Please also place any comments you may have, regarding any of the questions. For example - unable to use gauze swab square as patient retching or if an alternative form of airway protection was used such as micropore tape, please specify here, and clarify whether this was used effectively for the duration of the procedure.
- To ensure patient safety was maintained throughout the procedure, please also tick the relevant yes or no boxes for whether you needed to intervene for questions one to five.
- Please note: the entire procedure is defined as PMC try-in to cementation, where crown removal or adjustment is not required.

Airway protection questions	Please Tick			Did you have to intervene for patient safety? Please Tick	
	Yes	No	Any Comments	Yes	No
1. Was the patient sat upright for the entire procedure? (By upright we mean that the child is not reclined by more than 45 degrees in the dental chair)					
2. Was the airway protected with either a gauze swab square or a Micro-Stix applicator for the entire procedure?					
3. If the gauze swab square was utilised, was it positioned correctly for the entire procedure? (By positioned correctly we mean it should extend to the palate and round the back of the mouth, so it completely covers the airway)					
4. If the Micro-Stix applicator was utilised, was it placed on the buccal aspect of the crown?					
5. If the Micro-Stix applicator was utilised, was it effective for the entire procedure? (By effective we mean that the Micro-Stix applicator					

remained securely attached to the PFMC for the entire procedure)					

2. Airway protection protocol

Please can you disseminate the following protocol to the Stage 3 BSc, Stage 4 and 5 BDS students on airway protection, during the placement of a PMC:

- It is essential that we protect the airway during the placement of a PMC on a child patient. We are placing small crowns on little people, who like to wriggle around and shut their mouths whenever they feel like it. Ultimately, we never know when a child is going to move during treatment but by following the method below, we will reduce the risk of a PMC being either aspirated or swallowed.

- When trying in or cementing a PMC on a child patient using the Hall Technique, you must use the following method of:
  1. Sit the child as upright as possible and if you need to put your patient back in the dental chair to visualise what you are doing, then do not recline your patient by no more than 45 degrees. For placing a PMC on a lower tooth, you may need to stand up or position yourself in front or to the side of your patient, to maintain direct vision.
     For upper teeth, it's trickier to maintain the optimum operator working position and you will need to recline the chair to achieve this, but you must make sure that your patient is not reclined by more than 45 degrees.

  2. As well as sitting the child upright, you must also protect the airway by using one of the following three methods:
     - Place a piece of gauze between the tongue and the tooth where the crown is to be fitted. The gauze should extend to the palate and round the back of the mouth, so it completely covers the airway.
     - Attach a Micro-Stix applicator (otherwise known as a sticky stick) to the buccal aspect of the PMC- this is the method that we use most often on clinic.
     - Secure a piece of micropore tape to the occlusal surface of the PMC. We tend to not use this method on clinic but it's important that you are aware of this method.

- Sticky sticks seem to lose their effectiveness between PMC try in and placement, if the same sticky stick is used to try in all the different sizes of crowns, they lose their stickiness. Therefore, please use one sticky stick per PMC. So, if you are wanting to try in three different sizes, then use one sticky stick on each PMC. If the sticky stick looks like it's about to come off at any time, then attach a new sticky stick to the PMC. Sticky sticks also seem to lose their effectiveness when they encounter any saliva, we obviously can't change the oral environment, but make sure that your gloves and the PMC are dry, before securing the sticky stick onto the buccal surface.

- Do not remove your airway protection until your clinician tells you that the PMC is seated in the correct position and that it is safe to do so. If you remove your airway protection before

the PMC is seated in the correct position, then your supervising clinician will have to remove and reseat the PMC without any method of airway protection.

- For any PMC that you place on a patient, please record your form of airway protection in the clinical notes, using the airway protection sticker. Please show them the sticker and advise them how they need to complete this.

3. Airway protection sticker

PMC – Airway Protection

If you are unable to protect the airway – **STOP** what you are doing and discuss with clinical supervisor.

Tooth to be crowned: ……………………..…….

Crown used, including size: ……………………

Patient reclined by no more than 45 degrees for entire procedure *Please circle* **YES / NO**

Method of airway protection for entire procedure

Tick all that apply ☐ Micro-stix applicator

☐ Gauze

☐ Micropore tape
